# Newborn dried blood spot samples in Denmark: the hidden figures of secondary use and research participation

**DOI:** 10.1038/s41431-018-0276-2

**Published:** 2018-10-04

**Authors:** Francisca Nordfalk, Claus Thorn Ekstrøm

**Affiliations:** 1 Center for Medical Sciences and Technologies, Section for Health Services Research, Department of Public Health, University of Copenhagen, Copenhagen, Denmark; 2 Section of Biostatistics, Department of Public Health, University of Copenhagen, Copenhagen, Denmark

**Keywords:** Ethics, Genetic services, Genetics research

## Abstract

Each year millions of newborns are part of a newborn disease-screening program in which, after initial screening, the newborn dried blood spot (NDBS) samples can be stored and used as a population-based research resource. However, very little knowledge exists about how these samples are used for secondary purposes. Our objective is to estimate and describe the usage of a NDBS-based national population biobank for secondary research purposes. We therefore conducted a scoping study with a literature search for all published articles using samples from the Danish Newborn Screening Biobank. Our main inclusion criteria were that the articles had to have actively used and analyzed one or more of the Danish NDBS samples for a purpose beyond the primary screening. Our search led to a final 104 articles, which were coded for three main purposes: (1) how many samples were used in each article, (2) the field of their research, and (3) information on consent and ethics approval as research. From our analysis, we present two main findings: an estimated use of up to 37.5% of all samples in the newborn screening biobank have been part of published research, and a shift in the research areas from methodological and metabolic studies to studies concerning mental illness. This paper provides new insights into the use of a national biobank, and we hope that the results will contribute to the discussions on the use of biological samples for research purposes, and also inspire a greater transparency in the future use of NDBS samples.

## Introduction

Newborn dried blood spots (NDBS) are routinely stored and used for research purposes. Every year, millions of newborns worldwide are a part of a newborn screening program, where a few drops of blood from the baby’s heel are collected on a filter paper card. After the primary screening program, the NDBS can be stored in a freezer at the Danish Newborn Screening Biobank, under the Danish Statens Serum Institute and used for secondary purposes.

With this paper, our aim is to move beyond the primary screening purpose and elucidate how often and for which objectives the Danish NDBS samples are used for secondary purposes. The aims of our study are (1) to estimate how often the Danish NDBS samples are used for secondary purposes and what the probability is of a person’s sample being used, and (2) to describe what kind of research the Danish NDBS samples are used for. From contact with the Danish Newborn Screening Biobank, we understood that no metadata exists on how many samples had been used for secondary purposes; nor for what kind of research the Danish NDBS samples had been used. To answer these questions, we therefore conducted a scoping study [1, 2] of all articles using the Danish NDBS samples.

We argue for the importance of reaching, as accurately as possible, an estimate of the use of samples in the Danish Newborn Screening Biobank from four main perspectives: First, the debates around the use of NDBS samples has often been concerning consent policies [3–6]. Here, it is crucial to understand the practical implications of seeking and giving consent to research based on the NDBS sample, yet no information regarding this is available. Without any numbers, these discussions are based on uncertain assumptions. Second, knowing what kind of research is conducted using the NDBS samples is essential to understand both what the population might (unknowingly) be participating in, and what kind of research is made possible with the NDBS samples. Third, international debate has revolved around the relationship between gathering, storing, and using samples in biobanks—in particular, the importance of exploiting the full potential of the biobanks by sharing and using the samples [7]. Again, empirical insights to the actual use of biobanks would straighten these discussions. Information as to the relationship between the storage and use of samples from the Danish Newborn Screening Biobank will provide valid input in discussion on the use of biobank material and will serve as input to the debate of informed consent. And fourth, increased knowledge about when and how the Danish NDSB samples are used for research can help increase the level of understanding of why the NDBS samples are collected and stored for the public [8].

The use of NDBS samples for research purposes has also been a topic for international controversy. Most famous is the case from Texas where the dispute over the retention and use of the samples without parental knowledge or consent led to the destruction of over five million NDBS samples [9].

In Denmark, newborn screening was implemented nationally in 1982 [10], and since then an average of 62,000 newborns are annually a part of the Danish newborn screening program. Although the screening program is legally voluntary, it might not always be perceived as such by the parents. The blood spots are sent from the hospitals to the national Statens Serum Institute [10] where they are currently screened for 17 diseases [11] and subsequently stored indefinitely in the Danish Newborn Screening Biobank at the institute [12]. The samples are stored for three reasons: for the use of the child and the family, in case of further diagnostics or for identification purposes; for use in quality assurance and development of new analysis methods; and finally, the biobank also serves as a national resource for research purposes [13]. By Danish law, the samples can legally be stored and later used without explicit informed consent [14]; accordingly, problems of selection bias are avoided. Consent for the storage and subsequent use of the samples is embedded in allowing the sample to be taken. The policies enabling this kind of population-based data sourcing and usage are not unique in the Danish setting but are in many ways an example of Denmark as a “research radical country” [15]. In practice, consenting to having your child screened for diseases is also a (tacit) consent both to having your child’s sample stored and also to the sample being used for secondary purposes [16]. Acquiring access to the NDBS samples for researchers is a four-step process. First, the project must be approved by a Research Ethics Committee and the Danish Data Protection Agency. Second, the project may apply via Scientific Services; the joint Danish port of access to biological material and data under the Danish Health Data Authority. The application will then be forwarded to the Coordinating Centre at the Danish National Biobank, which will process the case. Third, all applications received are processed and accessed by the Scientific Board of the Danish National Biobank. And finally, if the three steps above are all approved and the terms of the hand-out are agreed upon, the samples are then retrieved and handed out [17].

Opting out of the research participation by signing up in the “use-of-tissue-register” [vævsanvendelsesregisteret] [18] is a possibility in Denmark; however, fewer than 500 people (from a population of ~ 5.7 million) are currently in the “use-of-tissue-register” [19]. It is unknown whether this is because people genuinely wish to participate in research based on their tissue sample, or because they are simply unaware of the possible participation and the opt-out option [20]. Moreover, the Danish NDBS samples can be linked to register data and to health records through the Central Person Register (known as CPR) number, a unique 10-digit civil registration number assigned to all the country’s residents [21, 22]. Similar to other Scandinavian countries, this creates a unique setting for epidemiologic research. The continuing national sampling and storage of NDBS from the whole of the Danish population thus makes a valuable resource for existing and future genomic research projects.

## Material and methods

In this scoping study [1, 2], we identified relevant studies by searching for articles using the Danish NDBS samples. The intention was originally to analyze how often these samples were used for research purposes. However, differentiating between what can be categorized as research and what is not research appeared less meaningful than we first expected. In this article, we therefore consider all purposes that are not the primary screening to be secondary purposes and will discuss their relation to research. Therefore, our main inclusion criteria were that the published articles had to have actively used and analyzed one or more of the Danish NDBS samples for a purpose beyond the primary screening.

We searched for articles in Scopus and PubMed MEDLINE as the two largest databases covering medicine and health-related research fields [23, 24]. We made a final search in both databases on 01 January 2018.

We combined the following terms in our search:

("Newborn" OR "Infant" OR "neonatal”) AND ("Bloodspot*" OR "Blood spot*" OR "NDBS" OR "DBS" OR "DBSS" OR "PKU" OR "Guthrie card") AND ("Denmark" OR "Danish").

In addition, a search for articles directly referring to the Danish Newborn Screening Biobank was undertaken with the following search string:

(“Danish Newborn Screening Biobank”) OR (“DNSB”)

**Fig. 1:**
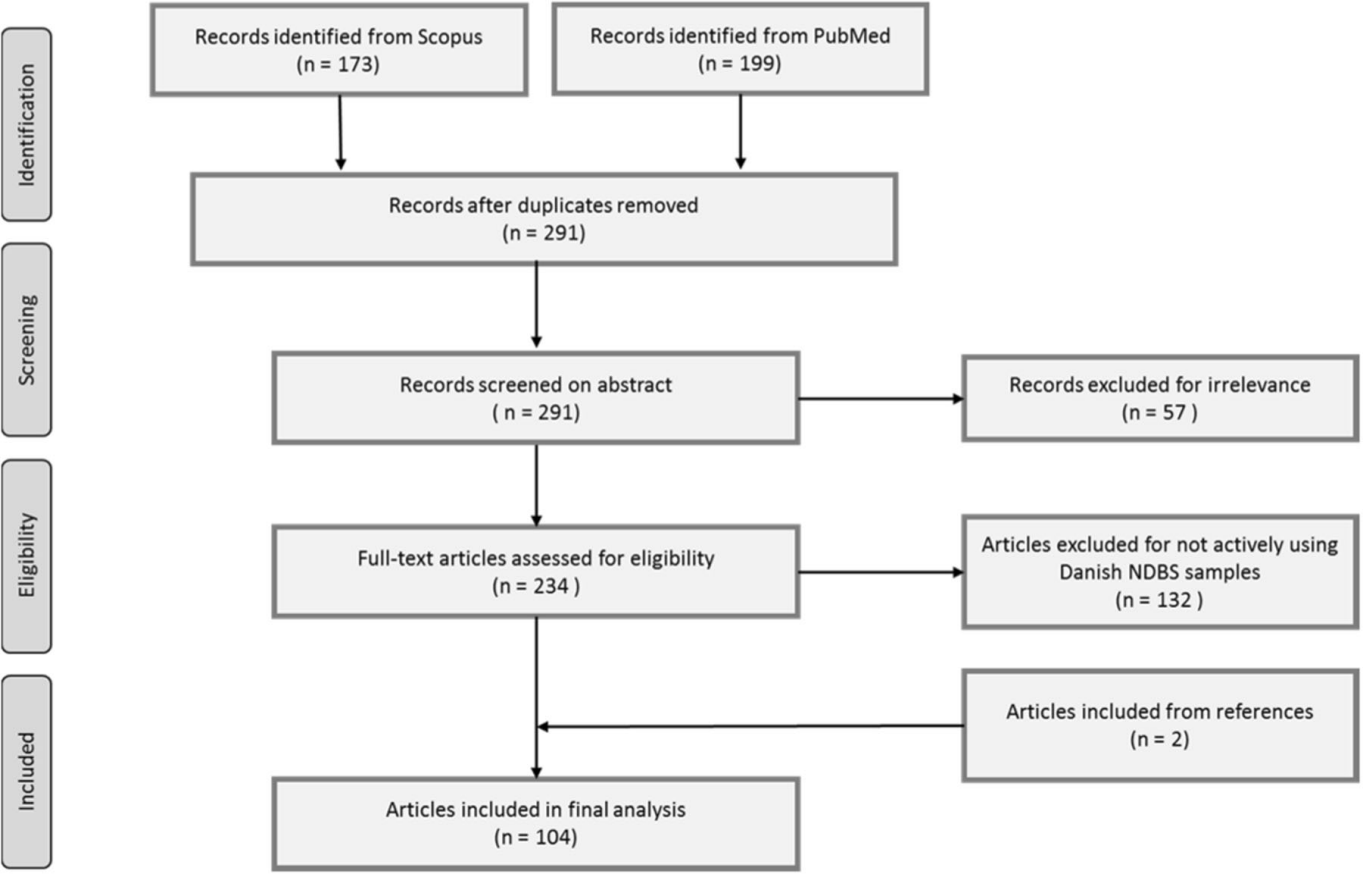
Prisma Flow on search

Our initial search resulted in 372 published articles. Removing duplicates left 291 individual articles. These we screened on abstract for relevance. Exclusion criteria based on abstract mainly included: reviews; studies on attitudes; other use of the abbreviations in our search; and articles from before 1982. The remaining 234 articles were screened on full text for eligibility. The main exclusion criteria were as follows: if the articles did not actively use Danish NDBS samples, but were, for the majority, studies with non-Danish samples; legal, social, policy, ethical, or future perspectives of NDBS samples and biobanking; studies based on other neonatal blood tests; or studies on phenylketonuria without the use of NDBS samples. This left 102 articles. All of these articles were screened on references for additional articles using Danish NDBS samples. This resulted in another two articles being included in our study. Finally, this gave us 104 published articles (please full list of articles in the supplementary material). The flow is presented in Fig. 1.

All of the final 104 eligible articles were combined in one Excel data set for later analysis in R. Nvivo 11 Pro was used for all coding of the articles. We coded all 104 articles for the following three main purposes: (1) how many samples were used in each article, (2) the field of their research, and (3) information on consent and ethics approval for research.

The number of samples used was recorded manually by detecting and coding all the samples in each of the 104 articles. We searched for the number of samples, whether the samples were a clinical case, cases (patients), controls, cohort, or a random sampling. In order to give an estimate of how many Danish NDBS samples have been used for research purposes in total, we sought to eliminate counting the reuse of samples. Approximately 80,000 Danish NDBS samples were included in a large research project, iPSYCH, a Danish population-based case–cohort, aimed at unraveling the genetic and environmental structure of five mental disorders (autism, ADHD, schizophrenia, bipolar disorder, and depression) [25], resulting in multiple published articles. All samples in articles from the iPSYCH project were therefore counted within this total. Further, some samples were used for more than one article. In cases where this was stated, the samples were only counted in the first article.

## Results

### Estimations of use of the Danish NDBS samples

The first article using the Danish NDBS samples is from 1991 and the latest is from November 2017. There is an increase in the number of articles throughout the period, which is not surprising considering the expansion of possibilities of using the NDBS samples. With the increase in articles there is also an increase in the number of samples used, and the probability for one person’s sample having been used therefore also increases over time. We have estimated the maximum proportion of NDBS samples that have been used for secondary purposes in published manuscripts by extracting the number of samples used in all articles surveyed divided by the cumulative number of newborns since the beginning of the newborn screening program. The 104 articles had an estimated total use of 794,157 individual samples. The State Serum Institute was not able to provide the exact number of samples in their biobank, but based on earlier literature, the biobank holds samples from ~ 95% of all births [19, 25]. The number of samples in the biobank from 1982–2017, therefore came to 2,120,163 at the time of our analysis. This would suggest that an estimated average of 37.5% of the samples in the Danish newborn screening biobank have therefore been used for secondary purposes, the research outcomes of which were published in an academic journal by 1 January 2018. As we cannot be certain that there are no duplicate individuals among the 794,157 samples, the estimate provides an upper limit for the proportion of individuals in the Danish Newborn Screening Biobank who have had their samples used for research.

**Fig. 2:**
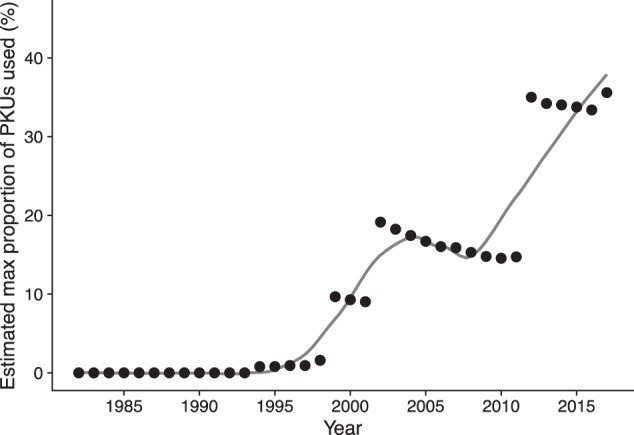
Estimated proportion of NDBS samples used for secondary research as a function of year (the black dots). The points indicate the cumulative prevalence, whereas the solid curve shows a smoothed trend line

The estimates in Fig. 2 are based on the number of samples found to have been used each year, as recorded in the published articles. The estimates presented here account for the use to date based on published research articles. The true percentage of individuals in the Danish newborn screening biobank who have been used for secondary research purposes could be much higher as we do not have any information on usage that is still in the publication pipeline or individuals who have been used for research that is not published. Estimations of future use are somewhat unpredictable. We know that the number of samples entering the biobank is closely linked to the number of births. However, the number of samples used for research purposes is dependent on a number of unpredictable factors, such as the interest in these samples compared with others, possibilities for funding for research projects using the NDBS samples, and policies concerning the use of Danish NDBS samples.

Of the 104 articles, 75 identify as research projects and therefore have an approval from the relevant research ethics committee. Eleven articles declare that the project does not constitute a health-related research project but is considered a developmental project for the Newborn Screening Program instead and therefore does not require a separate approval from the research ethics committee. And 18 of the articles do not clearly state anything about research or approvals from an ethics committee. Finally, only 16 of the articles clearly state having consent from all of their participants. However, it is not always precisely stated how consent is understood, nor how it was obtained.

### Research fields

**Fig. 3:**
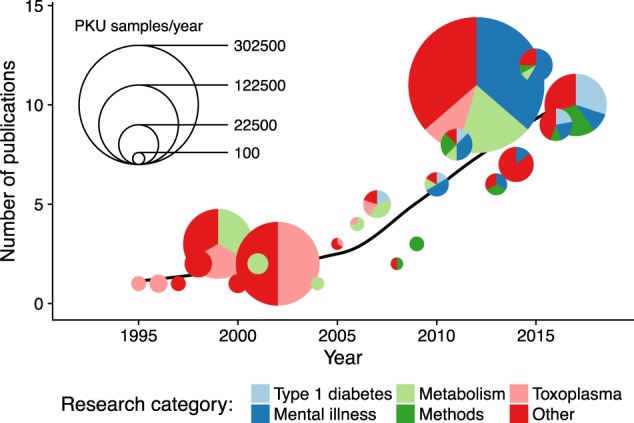
All use of Danish NDBS samples over time. Each bubble shows the number of NDBS articles per year, the color distribution indicates the relative contributions of the articles to the various research fields, whereas the bubble sizes indicate the total number of samples involved in the articles from that year. The black curve is a smoothed trend curve of article frequencies

The Danish NDBS samples have been used to facilitate studies of a variety of fields. By categorizing each article, we found that the rates at which the NDBS samples are being utilized for secondary research publication have varied substantially over time and research field, as shown in Fig. 3.

Approximately one-third (*n* = 36) of the articles study the methodological possibilities of NDBS samples. Of those, 14 articles study the methodological approach to using NDBS samples for a specific diagnosis, and 13 of the articles study methods without targeting a specific disease. Besides the methodological studies, the largest research field in the articles is mental illness. Twenty-three out of the 104 articles, and an estimated 91,162 individual samples, have been used to study either a specific exposure with mental illness as outcome, or the possible genetic composition found in children or adults with a mental illness. Other larger research fields include diseases related to the metabolism (15 articles), type 1 diabetes (9 articles), and toxoplasma (8 articles). The fields of metabolism and toxoplasma are a current or former part of the primary newborn screening program; however, we have only included articles that study these fields in a setting secondary to the primary screening.

Of the 794,157 used samples, a relatively large number, 84,299, are from participants with a diagnosis. The possibility of participating in research therefore is higher for people with a diagnosis (which often only comes about when individuals reach childhood or adulthood). For instance, ~ 12,000 people born after 1982 are diagnosed with type 1 diabetes (calculation based on [26]). We found that 7509 individual NDBS samples from people with a type 1 diabetes diagnosis have been used for research purposes. This would suggest that an estimated 62% of all samples from patients with type 1 diabetes have been used for research purposes, an increase of 25 percentage points compared with the overall proportion found for the individuals with a Danish NDBS samples.

As shown in Fig. 3, the numbers of samples used vary widely from year to year. As for research fields, there is an early interest in toxoplasma, which was a part of the Danish newborn screening program from 1999 to 2007 [11]. The later years have shown a marked increase in two main areas: type 1 diabetes, and more especially in mental illness. This could suggest a shift in the kind of research the samples are used for: moving from objectives closely related to the primary purpose, to objectives completely unrelated to the primary purpose but the research fields shown in Fig. 3 are purely descriptive and do not provide any indication as to which research fields will be the main in the future.

For all of the 104 articles, the top three most productive authors are Hougaard, D. (first author or co-author of 53 articles), Hollegaard, M. (first author or co-author of 29 articles), and Nørgaard-Pedersen, B. (first author or co-author of 27 articles). All three authors are current or former employees at the State Serum Institute.

## Discussion

Denmark is known as the “epidemiologist’s dream” [27] because of its widespread register structure and vast data collection [28]. A national biobank containing samples from a majority of the population since 1982 hugely expands the opportunities for population-wide research using both registers and genetic information. However, information about the usage of this resource is non-existent for both researchers and for the public, who contributed to it. This paper is the first attempt to elucidate how this national biobank has been utilized.

From the analysis of all articles using the Danish NDBS samples, our findings indicate that just under 40% of the samples in the Newborn Screening Biobank have been used for secondary purposes. Since this is, to our knowledge, the first ever actual estimate of the secondary use of a national biobank, it is not possible to compare it with the use of other biobanks. Furthermore, the earliest article using the Danish NDBS samples is from 1991, almost 10 years after the first sample was stored. This could indicate that the storing of the samples was initially not with a research purpose in mind.

Any research project using the samples must prove that the project has been approved by the Danish Data Protection Agency, and by the Scientific Ethical Committee system, as well as by the Steering Committee for Scientific Use of the Danish Newborn Screening Biobank [12]. According to Danish law [14], all health-related research projects require informed consent from each participant. However, because using the Danish NDBS samples for research purposes is considered to be register-based research [29], the National Committee on Health Research Ethics can waive the requirement for consent, if the research project does not imply health-related risks, and if the research project does not in other ways burden the participant. An exception can also be made if it would be impossible or disproportionately difficult to acquire consent [14]. As both are often the case for research projects using the Danish NDBS samples, projects are often permitted by The National Committee on Health Research Ethics to be implemented without consent. However, in the light of the practical implications for consent, our findings suggest that just under 40% of the population born in Denmark after 1982 have had their sample used for purposes beyond the primary screening. If researchers had to actively seek consent for the use of each sample for secondary purposes, it would have implied getting consent from the estimated 794,157 individuals in the period from 1991 to 2017. It is unknown how many people would not participate in research based on their NDBS samples, if they had to give consent for each project. However, one of the research projects using almost 100,000 NDBS samples incorporated consent when the samples were taken [30]. Here, only 179 (0.18%) declined participation. In research projects using fresh NDBS samples, this could prove a way to get explicit consent for the specific study. However, this solution will not be possible for samples already stored in the biobank. In these cases, obtaining consent could prove much more problematic. Moreover, people who have a diagnosis of interest for researchers using the Danish NDBS samples, have a higher probability of having their sample used, as exemplified with type 1 diabetes. Having a diagnosis of interest would therefore imply having to give consent more often.

Our findings further indicate a shift in the type of research carried out using the Danish NDBS samples. What was in the beginning, a resource for research projects closely related to the primary purpose, is now also a resource for research with objectives not in any way related to the primary purpose. This shift could represent a change in how the researchers perceive the Danish Newborn Screening Biobank as a resource, and it could also become crucial for how the secondary use of NDBS samples is perceived by the parents of the newborns and by the public at large.

One field particularly worth discussing is the usage of NDBS samples for studying mental illness. Twenty-three of the 104 articles use the Danish NDBS samples to study mental illness, which shows a descriptive trend in how the samples have been used until now. These studies further show how important a resource the NDBS biobank is. It is well established that it can be difficult to get people to participate in research on mental illness [31] and using the NDBS samples linked with information about mental illness must be considered a valuable resource for increasing knowledge on mental illness and possible treatment. These studies are therefore often done without explicit consent from the participants. This serves as an example of the complex situation of using the NDBS samples without consent: it facilitates vital research that could otherwise not be done, and simultaneously requires people to participate in research they might not wish to participate in.

Even though the majority of the articles included are defined as research, some of the included studies could be considered “non-research”. For instance, some of the methodological studies presented in the articles are considered a developmental project for the Newborn Screening Program. However, none of the samples were gathered with the primary purpose of being a part of a methodological study. Moreover, not all methodological articles are considered a developmental project for the Newborn Screening Program. For instance, an article from 2009 by Hollegaard et al. [32] studying “High-Throughput Genotyping on Archived Dried Blood Spot Samples” is approved by the Danish Regional Ethics Committee (as a research project) whereas Poulsen et al. from 2015 [33] studying “High-Quality Exome Sequencing of Whole Genome Amplified Neonatal Dried Blood Spot DNA” is not considered research. So, differentiating between what is research and what is not, seems challenging for the researchers themselves. Likewise, the Newborn Screening Biobank has earlier stated how projects using biobank material should be considered “research projects” [12]. Finally, the Danish public would probably in many cases not recognize the difference between having their sample used for whole-genome amplification of DNA [34] as a part of a developmental project as opposed to a research study of levels of vitamin D [35]. This further indicates that the division between what is research and what is not, might not be clear-cut, neither in practice nor in theory.

We also found that the main users of the NSBS samples are employed at the State Serum Institute. This could indicate that the knowledge of the availability of the samples as a resource is, for the general Danish research community, rather limited; or that the procedures for acquiring access to the samples are complicated; or that research involving the Danish NDBS samples is often done in collaboration with the specialized researchers at the State Serum Institute. In any case, it could be considered problematic that samples belonging to the whole population are most often only used by those controlling the very biobank that holds the samples.

Returning to our own study, several factors could influence how close our estimates are to the actual number of Danish NDBS samples used for research purposes. First of all, our search led us to the 104 articles. We are aware that some articles using the Danish NDBS samples might not appear from this search, if they do not state that their results are based on these samples. Not clearly stating where one’s data are originated does in itself bring about challenges, and much work is being done in order to facilitate proper citation of bio resources [36]. We have tried to minimize the risk of unfound articles by searching two different databases and by checking the references of each found article. Second, it is possible that samples have been used for research purposes, but that this use has not been reported in academic articles. The extent of this usage is unknown. Moreover, we can only make our estimates based on the number of samples reported in the articles, which might not always equal the actual number of samples withdrawn for each project. Finally, we have tried to identify how many individual samples have been used, though it is possible that a sample is utilized for more than one research project, without this being stated in the articles. However, bearing in mind that there should always be a part of the NDBS sample left for the child, the probability of multiple usage of the same sample decreases. With that said, we believe our estimate to be solidly-based in the circumstances. It is, to our knowledge, the first and only estimate of the actual usage of a national biobank, and therefore the best available estimate of the use of NDBS samples for secondary purposes.

## Conclusions

With this paper, we have presented new insights into the use of a national biobank. From our analysis we present two main findings. First, there has been an estimated use of almost 800,000 Danish NDBS samples, corresponding to ~ 37.5% of all samples in the biobank based on published manuscripts alone. Further, we clarified how these samples have been used principally for methodological studies or studies concerning mental illness, and how there has been a shift away from using the samples for studies closely related to the primary purpose, toward studies not related to the primary purposes. The only way for us to acquire this knowledge was to go through all articles that report use of the samples, as very little information was otherwise accessible. We believe that the information presented in this paper is important not just as an empirical finding for the discussions concerning the use of biological samples for secondary purposes, but also as information for the general public. As the samples are taken from almost all children born in Denmark, it is a national biobank, and the information derived from the samples should arguably therefore also belong to the nation. If knowledge about the use of the Danish NDBS samples is not shared, we risk creating a sense of uncertainty and possibly even mistrust and thereby risk that parents will in future refuse to have their child’s sample stored. The need for transparency for the data subject as likewise an essential notion of the European Union’s general data protection regulation (REF) [37], which increases the need for these considerations for the Danish Newborn Screening biobank and biobanks in general. We hope that the results presented in this paper will contribute to discussions around the use of biological samples for research purposes, and also that we will have led the way towards a greater transparency in the future use of NDBS samples and biobank samples in general.

## Electronic supplementary material

Supplementary material for “Newborn Dried Blood Spot Samples in Denmark: The Hidden Figures of Secondary Use and Research Participation”: 104 articles
