# Supplementary material for: Newborn dried blood spot samples in Denmark: the hidden figures of secondary use and research participation
Source: Eur J Hum Genet. 2018 Oct 4;27(2):203–10. doi: 10.1038/s41431-018-0276-2 (PMC6336790; doi:10.1038/s41431-018-0276-2)
Supplement: Supplementary file 1 — Supplementary material for “Newborn Dried Blood Spot Samples in Denmark: The Hidden Figures of Secondary Use and Research Participation”: 104 articles [file 41431_2018_276_MOESM1_ESM.docx]

**Supplementary material for “Newborn Dried Blood Spot Samples in Denmark: The Hidden Figures of Secondary Use and Research Participation”: 104 articles**

1. Kyvsgaard, J.N., Overgaard, A.J., Thorsen, S.U., Hansen, T.H., Pipper, C.B., Mortensen, H.B., Pociot, F., Svensson, J. (2017). High Neonatal Blood Iron Content Is Associated with the Risk of Childhood Type 1 Diabetes Mellitus. Nutrients, 9(11), 1221.
2. Pedersen, C.B., Bybjerg-Grauholm, J., Pedersen, M.G., Grove, J., Agerbo, E., Bækvad-Hansen, M., Poulsen, J.B., Hansen, C. S., McGrath, J.J., Als, T. D., Goldstein, J. I., Neale, B.M., Daly, M.J., Hougaard, D.M., Mors, O., Nordentoft, M., Børglum, A.D., Werge, T., Mortensen, P.B. (2017). The iPSYCH2012 case-cohort sample: new directions for unravelling genetic and environmental architectures of severe mental disorders. Molecular psychiatry, 23(1), 6.
3. Händel, M. N., Frederiksen, P., Cohen, A., Cooper, C., Heitmann, B. L., Abrahamsen, B. (2017). Neonatal vitamin D status from archived dried blood spots and future risk of fractures in childhood: results from the D-tect study, a population-based case-cohort study. The American journal of clinical nutrition, 106(1), 155-161.
4. Björkesten, J., Enroth, S., Shen, Q., Wik, L., Hougaard, D.M., Cohen, A.S., Sörensen, L., Giedraitis, V., Ingelsson, M., Larsson, A., Kamali-Moghaddam, M., Landegren, U. (2017). Stability of proteins in dried blood spot biobanks. Molecular & Cellular Proteomics, 16(7), 1286-1296.
5. Bækvad-Hansen, M., Bybjerg-Grauholm, J., Poulsen, J.B., Hansen, C.S., Hougaard, D.M., Hollegaard, M.V. (2017).Evaluation of whole genome amplified DNA to decrease material expenditure and increase quality. Molecular Genetics and Metabolism Reports, 11, pp. 36-45.
6. Thorsen, S.U., Pipper, C.B., Skogstrand, K., Pociot, F., Svensson, J. (2017). 25-hydroxyvitamin D and peripheral immune mediators: Results from two nationwide danish pediatric cohorts. Nutrients, 9 (4), art. no. 365.
7. Thorsen, S.U., Pipper, C.B., Alberdi-Saugstrup, M., Nielsen, S., Cohen, A., Lundqvist, M., Thygesen, L.C., Ascherio, A., Svensson, J. (2017). No association between vitamin D levels around time of birth and later risk of developing oligo- and polyarticular juvenile idiopathic arthritis: a Danish case–cohort study. Scandinavian Journal of Rheumatology, 46 (2), pp. 104-111.
8. Bybjerg-Grauholm, J., Hagen, C.M., Khoo, S.K., Johannesen, M.L., Hansen, C.S., Bækvad-Hansen, M., Christiansen, M., Hougaard, D.M., Hollegaard, M.V. (2017). RNA sequencing of archived neonatal dried blood spots. Molecular Genetics and Metabolism Reports, 10, pp. 33-37.
9. Nielsen, N.M., Munger, K.L., Koch-Henriksen, N., Hougaard, D.M., Magyari, M., Jørgensen, K.T., Lundqvist, M., Simonsen, J., Jess, T., Cohen, A., Stenager, E., Ascherio, A. (2017). Neonatal Vitamin D status and risk of multiple sclerosis: A population-based case-control study. Neurology, 88 (1), pp. 44-51.
10. Thorsen, S.U., Pipper, C.B., Eising, S., Skogstrand, K., Hougaard, D.M., Svensson, J., Pociot, F. (2017). Neonatal levels of adiponectin, interleukin-10 and interleukin-12 are associated with the risk of developing type 1 diabetes in childhood and adolescence: A nationwide Danish case-control study. Clinical Immunology, 174, pp. 18-23.
11. Benros, M.E., Trabjerg, B.B., Meier, S., Mattheisen, M., Mortensen, P.B., Mors, O., Børglum, A.D., Hougaard, D.M., Nørgaard-Pedersen, B., Nordentoft, M., Agerbo, E. (2016). Influence of Polygenic Risk Scores on the Association Between Infections and Schizophrenia. Biological Psychiatry, 80 (8), pp. 609-616.
12. Knight, A.K., Craig, J.M., Theda, C., Bækvad-Hansen, M., Bybjerg-Grauholm, J., Hansen, C.S., Hollegaard, M.V., Hougaard, D.M., Mortensen, P.B., Weinsheimer, S.M., Werge, T.M., Brennan, P.A., Cubells, J.F., Newport, D.J., Stowe, Z.N., Cheong, J.L.Y., Dalach, P., Doyle, L.W., Loke, Y.J., Baccarelli, A.A., Just, A.C., Wright, R.O., Téllez-Rojo, M.M., Svensson, K., Trevisi, L., Kennedy, E.M., Binder, E.B., Iurato, S., Czamara, D., Räikkönen, K., Lahti, J.M.T., Pesonen, A.-K., Kajantie, E., Villa, P.M., Laivuori, H., Hämäläinen, E., Park, H.J., Bailey, L.B., Parets, S.E., Kilaru, V., Menon, R., Horvath, S., Bush, N.R., LeWinn, K.Z., Tylavsky, F.A., Conneely, K.N., Smith, A.K. (2016). An epigenetic clock for gestational age at birth based on blood methylation data. Genome Biology, 17 (1), art. no. 206.
13. Jacobsen, R., Thorsen, S.U., Cohen, A.S., Lundqvist, M., Frederiksen, P., Pipper, C.B., Pociot, F., Thygesen, L.C., Ascherio, A., Svensson, J., Heitmann, B.L. (2016). Neonatal vitamin D status is not associated with later risk of type 1 diabetes: results from two large Danish population-based studies. Diabetologia, 59 (9), pp. 1871-1881.
14. Thorsen, S.U., Jakobsen, C., Cohen, A., Lundqvist, M., Thygesen, L.C., Pipper, C., Ascherio, A., Svensson, J. (2016). Perinatal vitamin D levels are not associated with later risk of developing pediatric-onset inflammatory bowel disease: a Danish case-cohort study. Scandinavian Journal of Gastroenterology, 51 (8), pp. 927-933.
15. Poulsen, J.B., Lescai, F., Grove, J., Bækvad-Hansen, M., Christiansen, M., Hagen, C.M., Maller, J., Stevens, C., Li, S., Li, Q., Sun, J., Wang, J., Nordentoft, M., Werge, T.M., Mortensen, P.B., Børglum, A.D., Daly, M., Hougaard, D.M., Bybjerg-Grauholm, J., Hollegaard, M.V. (2016). High-quality exome sequencing of whole-genome amplified neonatal dried blood spot DNA. PLoS ONE, 11 (4), art. no. e0153253.
16. Nissen, J.B., Hansen, C.S., Starnawska, A., Mattheisen, M., Børglum, A.D., Buttenschøn, H.N., Hollegaard, M. (2016). DNA methylation at the neonatal state and at the time of diagnosis: Preliminary support for an association with the estrogen receptor 1, gamma-aminobutyric acid B receptor 1, and myelin oligodendrocyte glycoprotein in female adolescent patients with OCD. Frontiers in Psychiatry, 7 (MAR), art. no. 35.
17. Petersen, J.P., Ebbesen, F., Hollegaard, M.V., Andersson, S., Hougaard, D.M., Thorlacius-Ussing, O., Henriksen, T.B. (2016). UGT1A1 ∗ 28 genotypes and respiratory disease in very preterm infants: A cohort study. Neonatology, 109 (2), pp. 124-129.
18. Lundbo, L.F., Harboe, Z.B., Clausen, L.N., Hollegaard, M.V., Sørensen, H.T., Hougaard, D.M., Konradsen, H.B., Nørgaard, M., Benfield, T. (2016). Genetic Variation in NFKBIE Is Associated With Increased Risk of Pneumococcal Meningitis in Children. EBioMedicine, 3, pp. 93-99.
19. Kyvsgaard, J.N., Overgaard, A.J., Jacobsen, L.D., Thorsen, S.U., Pipper, C.B., Hansen, T.H., Husted, S., Mortensen, H.B., Pociot, F., Svensson, J. (2016). Low perinatal zinc status is not associated with the risk of type 1 diabetes in children. Pediatric Diabetes, 18 (7), pp. 637-642.
20. Grauholm, J., Khoo, S.K., Nickolov, R.Z., Poulsen, J.B., Bækvad-Hansen, M., Hansen, C.S., Hougaard, D.M., Hollegaard, M.V. (2015). Gene expression profiling of archived dried blood spot samples from the Danish Neonatal Screening Biobank. Molecular Genetics and Metabolism, 116 (3), pp. 119-124.
21. Thorup, A.A.E., Jepsen, J.R., Ellersgaard, D.V., Burton, B.K., Christiani, C.J., Hemager, N., Skjærbæk, M., Ranning, A., Spang, K.S., Gantriis, D.L., Greve, A.N., Zahle, K.K., Mors, O., Plessen, K.J., Nordentoft, M. (2015). The Danish High Risk and Resilience Study - VIA 7 - A cohort study of 520 7-year-old children born of parents diagnosed with either schizophrenia, bipolar disorder or neither of these two mental disorders. BMC Psychiatry, 15 (1), art. no. 233.
22. Dahlin, A.M., Hollegaard, M.V., Wibom, C., Andersson, U., Hougaard, D.M., Deltour, I., Hjalmars, U., Melin, B. (2015). CCND2, CTNNB1, DDX3X, GLI2, SMARCA4, MYC, MYCN, PTCH1, TP53, and MLL2 gene variants and risk of childhood medulloblastoma. Journal of Neuro-Oncology, 125 (1), pp. 75-78.
23. Schmock, H., Vangkilde, A., Larsen, K.M., Fischer, E., Birknow, M.R., Jepsen, J.R.M., Olesen, C., Skovby, F., Plessen, K.J., Mørup, M., Hulme, O., Baaré, W.F.C., Didriksen, M., Siebner, H.R., Werge, T., Olsen, L. (2015). The Danish 22q11 research initiative. BMC Psychiatry, 15 (1), art. no. 220.
24. Nielsen, P.R., Agerbo, E., Skogstrand, K., Hougaard, D.M., Meyer, U., Mortensen, P.B. (2015). Neonatal levels of inflammatory markers and later risk of schizophrenia. Biological Psychiatry, 77 (6), pp. 548-555.
25. Nordentoft, M., Larsen, J.T., Pedersen, C.B., Sørensen, H.J., Hollegaard, M.V., Hougaard, D.M., Mortensen, P.B., Petersen, L. (2015). Delay in blood sampling for routine newborn screening is associated with increased risk of schizophrenia. Schizophrenia Research, 162 (1-3), pp. 90-96.
26. Faurschou, S., Mouritsen, A., Johannsen, T.H., Hougaard, D.M., Cohen, A., Duno, M., Juul, A., Main, K.M. (2015). Hormonal disturbances due to severe and mild forms of congenital adrenal hyperplasia are already detectable in neonatal life. Acta paediatrica (Oslo, Norway : 1992), 104 (2), pp. e57-e62.
27. Agerbo, E., Sullivan, P.F., Vilhjálmsson, B.J., Pedersen, C.B., Mors, O., Børglum, A.D., Hougaard, D.M., Hollegaard, M.V., Meier, S., Mattheisen, M., Ripke, S., Wray, N.R., Mortensen, P.B. (2015). Polygenic risk score, parental socioeconomic status, family history of psychiatric disorders, and the risk for schizophrenia: A Danish population-based study and meta-analysis. JAMA Psychiatry, 72 (7), pp. 635-641.
28. Luo, X.-J., Mattheisen, M., Li, M., Huang, L., Rietschel, M., Børglum, A.D., Als, T.D., Van Den Oord, E.J., Aberg, K.A., Mors, O., Mortensen, P.B., Luo, Z., Degenhardt, F., Cichon, S., Schulze, T.G., Nöthen, M.M., Su, B., Zhao, Z., Gan, L., Yao, Y.-G. (2015). Systematic Integration of Brain eQTL and GWAS Identifies ZNF323 as a Novel Schizophrenia Risk Gene and Suggests Recent Positive Selection Based on Compensatory Advantage on Pulmonary Function. Schizophrenia Bulletin, 41 (6), pp. 1294-1308.
29. Winkel, B.G., Yuan, L., Olesen, M.S., Sadjadieh, G., Wang, Y., Risgaard, B., Jabbari, R., Haunsø, S., Holst, A.G., Hollegaard, M.V., Tfelt-Hansen, J., Jespersen, T. (2015). The role of the sodium current complex in a nonreferred nationwide cohort of sudden infant death syndrome. Heart Rhythm, 12 (6), pp. 1241-1249.
30. Lundbo, L.F., Sørensen, H.T., Clausen, L.N., Hollegaard, M.V., Hougaard, D.M., Konradsen, H.B., Harboe, Z.B., Nørgaard, M., Benfield, T. (2015). Mannose-binding lectin gene, MBL2, polymorphisms do not increase susceptibility to invasive meningococcal disease in a population of Danish children. Open Forum Infectious Diseases, 2 (4), art. no. ofv127.
31. Debost, J.-C., Petersen, L., Grove, J., Hedemand, A., Khashan, A., Henriksen, T., Mors, O., Hollegaard, M., Hougaard, D., Nyegaard, M., Børglum, A., Mortensen, P.B. (2015). Investigating interactions between early life stress and two single nucleotide polymorphisms in HSD11B2 on the risk of schizophrenia. Psychoneuroendocrinology, 60, pp. 18-27.
32. Petersen, J.P., Overvad, K., Hollegaard, M.V., Ebbesen, F., Henriksen, T.B., Thorlacius-Ussing, O., Hougaard, D.M., Schrøder, H. (2014). UGT1A1*28 polymorphism and acute lymphoblastic leukemia in children: a Danish case-control study. Pediatric research, 76 (5), pp. 459-463.
33. Petersen, J.P., Henriksen, T.B., Hollegaard, M.V., Vandborg, P.K., Hougaard, D.M., Thorlacius-Ussing, O., Ebbesen, F. (2014). Extreme neonatal hyperbilirubinemia and a specific genotype: A population-based case-control study. Pediatrics, 134 (3), pp. 510-515.
34. Lundbo, L.F., Harboe, Z.B., Clausen, L.N., Hollegaard, M.V., Sørensen, H.T., Hougaard, D.M., Konradsen, H.B., Nørgaard, M., Benfield, T. (2014). Mannose-binding lectin gene, MBL2, polymorphisms are not associated with susceptibility to invasive pneumococcal disease in children. Clinical Infectious Diseases, 59 (4), pp. e66-e71.
35. Jakobsen, C., Cleynen, I., Andersen, P.S., Vermeire, S., Munkholm, P., Paerregaard, A., Wewer, V. (2014). Genetic susceptibility and genotype-phenotype association in 588 Danish children with inflammatory bowel disease. Journal of Crohn's and Colitis, 8 (7), pp. 678-685.
36. Børglum, A.D., Demontis, D., Grove, J., Pallesen, J., Hollegaard, M.V., Pedersen, C.B., Hedemand, A., Mattheisen, M., Uitterlinden, A., Nyegaard, M., Ørntoft, T., Wiuf, C., Didriksen, M., Nordentoft, M., Nö then, M.M., Rietschel, M., Ophoff, R.A., Cichon, S., Yolken, R.H., Hougaard, D.M., Mortensen, P.B., Mors, O. (2014). Genome-wide study of association and interaction with maternal cytomegalovirus infection suggests new schizophrenia loci. Molecular Psychiatry, 19 (3), pp. 325-333.
37. Bønnelykke, K., Sleiman, P., Nielsen, K., Kreiner-Møller, E., Mercader, J.M., Belgrave, D., Den Dekker, H.T., Husby, A., Sevelsted, A., Faura-Tellez, G., Mortensen, L.J., Paternoster, L., Flaaten, R., Mølgaard, A., Smart, D.E., Thomsen, P.F., Rasmussen, M.A., Bonàs-Guarch, S., Holst, C., Nohr, E.A., Yadav, R., March, M.E., Blicher, T., Lackie, P.M., Jaddoe, V.W.V., Simpson, A., Holloway, J.W., Duijts, L., Custovic, A., Davies, D.E., Torrents, D., Gupta, R., Hollegaard, M.V., Hougaard, D.M., Hakonarson, H., Bisgaard, H. (2014). A genome-wide association study identifies CDHR3 as a susceptibility locus for early childhood asthma with severe exacerbations. Nature Genetics, 46 (1), pp. 51-55.
38. Englund, A., Rogvi, R.A., Melgaard, L., Greisen, G. (2014). Citrulline concentration in routinely collected neonatal dried blood spots cannot be used to predict necrotising enterocolitis. Acta Paediatrica, International Journal of Paediatrics, 103 (11), pp. 1143-1147.
39. Hollegaard, M.V., Grauholm, J., Nielsen, R., Grove, J., Mandrup, S., Hougaard, D.M. (2014). Archived neonatal dried blood spot samples can be used for accurate whole genome and exome-targeted next-generation sequencing. Molecular Genetics and Metabolism, 110 (1-2), pp. 65-72.
40. Abdallah, M.W., Mortensen, E.L., Greaves-Lord, K., Larsen, N., Bonefeld-Jørgensen, E.C., Nørgaard-Pedersen, B., Hougaard, D.M., Grove, J. (2013). Neonatal levels of neurotrophic factors and risk of autism spectrum disorders. Acta Psychiatrica Scandinavica, 128 (1), pp. 61-69.
41. Jacobsen, R., Abrahamsen, B., Bauerek, M., Holst, C., Jensen, C.B., Knop, J., Raymond, K., Rasmussen, L.B., Stougaard, M., Sørensen, T.I., Vaag, A.A., Heitmann, B.L. (2013). The influence of early exposure to vitamin D for development of diseases later in life. BMC Public Health, 13 (1), art. no. 515.
42. Hollegaard, M.V., Grauholm, J., Nørgaard-Pedersen, B., Hougaard, D.M. (2013). DNA methylome profiling using neonatal dried blood spot samples: A proof-of-principle study. Molecular Genetics and Metabolism, 108 (4), pp. 225-231.
43. Abdallah, M.W., Larsen, N., Grove, J., Bonefeld-Jørgensen, E.C., Nørgaard-Pedersen, B., Hougaard, D.M., Mortensen, E.L. (2013). Neonatal chemokine levels and risk of autism spectrum disorders: Findings from a Danish historic birth cohort follow-up study. Cytokine, 61 (2), pp. 370-376.
44. Hollegaard, M.V., Skogstrand, K., Thorsen, P., Nørgaard-Pedersen, B., Hougaard, D.M., Grove, J. (2013). Joint Analysis of SNPs and Proteins Identifies Regulatory IL18 Gene Variations Decreasing the Chance of Spastic Cerebral Palsy. Human Mutation, 34 (1), pp. 143-148.
45. Aksglaede, L., Garn, I.D., Hollegaard, M.V., Hougaard, D.M., Rajpert-De Meyts, E., Juul, A. (2012). Detection of increased gene copy number in DNA from dried blood spot samples allows efficient screening for Klinefelter syndrome. Acta Paediatrica, International Journal of Paediatrics, 101 (12), pp. e561-e563.
46. Abdallah, M.W., Larsen, N., Mortensen, E.L., Atladóttir, H.Ó., Nørgaard-Pedersen, B., Bonefeld-Jørgensen, E.C., Grove, J., Hougaard, D.M. (2012). Neonatal levels of cytokines and risk of autism spectrum disorders: An exploratory register-based historic birth cohort study utilizing the Danish Newborn Screening Biobank. Journal of Neuroimmunology, 252 (1-2), pp. 75-82.
47. Pedersen, M.G., Mortensen, P.B., Norgaard-Pedersen, B., Postolache, T.T. (2012). Toxoplasma gondii infection and self-directed violence in mothers. Archives of General Psychiatry, 69 (11), pp. 1123-1130.
48. Bross, P., Frederiksen, J.B., Bie, A.S., Hansen, J., Palmfeldt, J., Nielsen, M.N., Duno, M., Lund, A.M., Christensen, E. (2012). Heterozygosity for an in-frame deletion causes glutaryl-CoA dehydrogenase deficiency in a patient detected by newborn screening: Investigation of the effect of the mutant allele. Journal of Inherited Metabolic Disease, 35 (5), pp. 787-796.
49. Pedersen, C.B., Demontis, D., Pedersen, M.S., Agerbo, E., Mortensen, P.B., Børglum, A.D., Hougaard, D.M., Hollegaard, M.V., Mors, O., Cantor-Graae, E. (2012). Risk of schizophrenia in relation to parental origin and genome-wide divergence. Psychological Medicine, 42 (7), pp. 1515-1521.
50. Nyegaard, M., Demontis, D., Thestrup, B.B., Hedemand, A., Sørensen, K.M., Hansen, T., Werge, T., Hougaard, D.M., Yolken, R.H., Mortensen, P.B., Mors, O., Børglum, A.D. (2012). No association of polymorphisms in human endogenous retrovirus K18 and CD48 with schizophrenia. Psychiatric Genetics, 22 (3), pp. 146-148.
51. Andresen, B.S., Lund, A.M., Hougaard, D.M., Christensen, E., Gahrn, B., Christensen, M., Bross, P., Vested, A., Simonsen, H., Skogstrand, K., Olpin, S., Brandt, N.J., Skovby, F., Nørgaard-Pedersen, B., Gregersen, N. (2012). MCAD deficiency in Denmark. Molecular Genetics and Metabolism, 106 (2), pp. 175-188.
52. Agergaard, P., Olesen, C., Østergaard, J.R., Christiansen, M., Sørensen, K.M. (2012). The prevalence of chromosome 22q11.2 deletions in 2,478 children with cardiovascular malformations. A population-based study. American Journal of Medical Genetics, Part A, 158 A (3), pp. 498-508.
53. Agergaard, P., Olesen, C., Østergaard, J.R., Christiansen, M., Sørensen, K.M. (2012) Chromosome 22q11.2 duplication is rare in a population-based cohort of Danish children with cardiovascular malformations. American Journal of Medical Genetics, Part A, 158 A (3), pp. 509-513.
54. Fode, P., Larsen, A.R., Feenstra, B., Jespersgaard, C., Skov, R.L., Stegger, M., Fowler, V.G., Andersen, P.S. (2012). Genetic variability in beta-defensins is not associated with susceptibility to staphylococcus aureus bacteremia. PLoS ONE, 7 (2), art. no. e32315.
55. Agerbo, E., Mortensen, P.B., Wiuf, C., Pedersen, M.S., McGrath, J., Hollegaard, M.V., Nørgaard-Pedersen, B., Hougaard, D.M., Mors, O., Pedersen, C.B. (2012). Modelling the contribution of family history and variation in single nucleotide polymorphisms to risk of schizophrenia: A Danish national birth cohort-based study. Schizophrenia Research, 134 (2-3), pp. 246-252.
56. Borch, L., Lund, A. M., Wibrand, F., Christensen, E., Søndergaard, C., Gahrn, B., Hougaard, D.M., Andersen, B.S., Gregersen, N. & Olsen, R. K. J (2011). Normal levels of plasma free carnitine and acylcarnitines in follow-up samples from a presymptomatic case of carnitine palmitoyl transferase 1 (CPT1) deficiency detected through newborn screening in Denmark. In *JIMD Reports-Case and Research Reports, 2011/3* (pp. 11-15).
57. Demontis, D., Nyegaard, M., Buttenschøn, H.N., Hedemand, A., Pedersen, C.B., Grove, J., Flint, T.J., Nordentoft, M., Werge, T., Hougaard, D.M., Sørensen, K.M., Yolken, R.H., Mors, O., Børglum, A.D., Mortensen, P.B. (2011). Association of GRIN1 and GRIN2A-D With schizophrenia and genetic interaction with maternal herpes simplex virus-2 infection affecting disease risk. American Journal of Medical Genetics, Part B: Neuropsychiatric Genetics, 156 (8), pp. 913-922.
58. Mortensen, P.B., Pedersen, C.B., Mcgrath, J.J., Hougaard, D.M., Nørgaard-Petersen, B., Mors, O., Børglum, A.D., Yolken, R.H. (2011). Neonatal antibodies to infectious agents and risk of bipolar disorder: A population-based case-control study. Bipolar Disorders, 13 (7-8), pp. 624-629.
59. Janik, D.K., Lindau-Shepard, B., Nørgaard-Pedersen, B., Heilmann, C., Pass, K.A. (2011). Improved immunoassay for the detection of severe combined immunodeficiency. Clinical Chemistry, 57 (8), pp. 1207-1209.
60. Hollegaard, M.V., Grove, J., Grauholm, J., Kreiner-Møller, E., Bønnelykke, K., Nørgaard, M., Benfield, T.L., Nørgaard-Pedersen, B., Mortensen, P.B., Mors, O., Sørensen, H.T., Harboe, Z.B., Børglum, A.D., Demontis, D., Ørntoft, T.F., Bisgaard, H., Hougaard, D.M. (2011). Robustness of genome-wide scanning using archived dried blood spot samples as a DNA source. BMC Genetics, 12, art. no. 58.
61. Van Schijndel, J.E., Van Zweeden, M., Van Loo, K.M.J., Djurovic, S., Andreassen, O.A., Hansen, T., Werge, T., Nyegaard, M., Sørensen, K.M., Nordentoft, M., Mortensen, P.B., Mors, O., Børglum, A.D., Del-Favero, J., Norrback, K.-F., Adolfsson, R., Hert, M.D., Claes, S., Cichon, S., Rietschel, M., Nöthen, M.M., Kallunki, P., Pedersen, J.T., Martens, G.J.M. (2011). Dual association of a TRKA polymorphism with schizophrenia. Psychiatric Genetics, 21 (3), pp. 125-131.
62. Winkel, B.G., Hollegaard, M.V., Olesen, M.S., Svendsen, J.H., Haunsø, S., Hougaard, D.M., Tfelt-Hansen, J. (2011). Whole-genome amplified DNA from stored dried blood spots is reliable in high resolution melting curve and sequencing analysis. BMC Medical Genetics, 12, art. no. 22.
63. Eising, S., Nilsson, A., Carstensen, B., Hougaard, D.M., Nørgaard-Pedersen, B., Nerup, J., Lernmark, Å., Pociot, F. (2011). Danish children born with glutamic acid decarboxylase-65 and islet antigen-2 autoantibodies at birth had an increased risk to develop type 1 diabetes. European Journal of Endocrinology, 164 (2), pp. 247-252.
64. McGuire, J.N., Eising, S., Wägner, A.M., Pociot, F. (2010). Screening newborns for candidate biomarkers of type 1 diabetes. Archives of Physiology and Biochemistry, 116 (4-5), pp. 227-232.
65. McGrath, J.J., Eyles, D.W., Pedersen, C.B., Anderson, C., Ko, P., Burne, T.H., Norgaard-Pedersen, B., Hougaard, D.M., Mortensen, P.B. (2010). Neonatal vitamin D status and risk of schizophrenia: A population-based case-control study. Archives of General Psychiatry, 67 (9), pp. 889-894.
66. Mortensen, P.B., Pedersen, C.B., Hougaard, D.M., Nørgaard-Petersen, B., Mors, O., Børglum, A.D., Yolken, R.H. (2010). A Danish National Birth Cohort study of maternal HSV-2 antibodies as a risk factor for schizophrenia in their offspring. Schizophrenia Research, 122 (1-3), pp. 257-263.
67. Olsen, R.K.J., Dobrowolski, S.F., Kjeldsen, M., Hougaard, D., Simonsen, H., Gregersen, N., Andresen, B.S. (2010). High-resolution melting analysis, a simple and effective method for reliable mutation scanning and frequency studies in the ACADVL gene. Journal of Inherited Metabolic Disease, 33 (3), pp. 247-260.
68. Sørensen, K.M., Agergaard, P., Olesen, C., Andersen, P.S., Larsen, L.A., Østergaard, J.R., Schouten, J.P., Christiansen, M. (2010). Detecting 22q11.2 deletions by use of multiplex ligation-dependent probe amplification on DNA from neonatal dried blood spot samples. Journal of Molecular Diagnostics, 12 (2), pp. 147-151.
69. Nyegaard, M., Demontis, D., Foldager, L., Hedemand, A., Flint, T.J., Sørensen, K.M., Andersen, P.S., Nordentoft, M., Werge, T., Pedersen, C.B., Hougaard, D.M., Mortensen, P.B., Mors, O., Børglum, A.D. (2010). CACNA1C (rs1006737) is associated with schizophrenia. Molecular Psychiatry, 15 (2), pp. 119-121.
70. Hollegaard, M.V., Grauholm, J., Børglum, A., Nyegaard, M., Nørgaard-Pedersen, B., Ørntoft, T., Mortensen, P.B., Wiuf, C., Mors, O., Didriksen, M., Thorsen, P., Hougaard, D.M. (2009). Genome-wide scans using archived neonatal dried blood spot samples. BMC Genomics, 10, art. no. 297.
71. Hollegaard, M.V., Thorsen, P., Norgaard-Pedersen, B., Hougaard, D.M. (2009). Genotyping whole-genome-amplified DNA from 3- to 25-year-old neonatal dried blood spot samples with reference to fresh genomic DNA. Electrophoresis, 30 (14), pp. 2532-2535.
72. Hollegaard, M.V., Grove, J., Thorsen, P., Nørgaard-Pedersen, B., Hougaard, D.M. (2009). High-throughput genotyping on archived dried blood spot samples. Genetic testing and molecular biomarkers, 13 (2), pp. 173-179.
73. Sorensen, K.M., Andersen, P.S., Larsen, L.A., Schwartz, M., Scheuten, J.P., Nygren, A.O.H. (2008). Multiplex ligation-dependent probe amplification technique for copy number analysis on small amounts of DNA material. Analytical Chemistry, 80 (23), pp. 9363-9368.
74. Skogstrand, K., Hougaard, D.M., Schendel, D.E., Bent, N.-P., Sværke, C., Thorsen, P. (2008). Association of preterm birth with sustained postnatal inflammatory response. Obstetrics and Gynecology, 111 (5), pp. 1118-1128.
75. Eising, S., Svensson, J., Skogstrand, K., Nilsson, A., Lynch, K., Andersen, P.S., Lernmark, Å., Hougaard, D.M., Pociot, F., Nørgaard-Pedersen, B., Nerup, J. (2007). Type 1 diabetes risk analysis on dried blood spot samples from population-based newborns: Design and feasibility of an unselected case-control study. Paediatric and Perinatal Epidemiology, 21 (6), pp. 507-517.
76. Klamer, A., Skogstrand, K., Hougaard, D.M., Nørgaard-Petersen, B., Juul, A., Greisen, G. (2007). Adiponectin levels measured in dried blood spot samples from neonates born small and appropriate for gestational age. European Journal of Endocrinology, 157 (2), pp. 189-194.
77. Lund, A.M., Joensen, F., Hougaard, D.M., Jensen, L.K., Christensen, E., Christensen, M., Nørgaard-Petersen, B., Schwartz, M., Skovby, F. (2007). Carnitine transporter and holocarboxylase synthetase deficiencies in The Faroe Islands. Journal of inherited metabolic disease, 30 (3), pp. 341-349.
78. Ostergaard, E., Hansen, F.J., Sorensen, N., Duno, M., Vissing, J., Larsen, P.L., Faeroe, O., Thorgrimsson, S., Wibrand, F., Christensen, E., Schwartz, M. (2007). Mitochondrial encephalomyopathy with elevated methylmalonic acid is caused by SUCLA2 mutations. Brain, 130 (3), pp. 853-861.
79. Mortensen, P.B., Nørgaard-Pedersen, B., Waltoft, B.L., Sørensen, T.L., Hougaard, D., Torrey, E.F., Yolken, R.H. (2007). Toxoplasma gondii as a Risk Factor for Early-Onset Schizophrenia: Analysis of Filter Paper Blood Samples Obtained at Birth. Biological Psychiatry, 61 (5), pp. 688-693.
80. Tvedegaard, K.C., Rüdiger, N.S., Pedersen, B.N., Møller, J. (2006). Detection of MTRR 66A→G polymorphism using the real-time polymerase chain reaction machine LightCycler for determination of composition of allele after restriction cleavage. Scandinavian Journal of Clinical and Laboratory Investigation, 66 (8), pp. 685-694.
81. Pedersen, C.B., Bischoff, C., Christensen, E., Simonsen, H., Lund, A.M., Young, S.P., Koeberl, D.D., Millington, D.S., Roe, C.R., Roe, D.S., Wanders, R.J.A., Ruiter, J.P.N., Keppen, L.D., Stein, Q., Knudsen, I., Gregersen, N., Andresen, B.S. (2006). Variations in IBD (ACAD8) in children with elevated C4-carnitine detected by tandem mass spectrometry newborn screening. Pediatric Research, 60 (3), pp. 315-320.
82. Madsen, P.P., Kibæk, M., Roca, X., Sachidanandam, R., Krainer, A.R., Christensen, E., Steiner, R.D., Gibson, K.M., Corydon, T.J., Knudsen, I., Wanders, R.J.A., Ruiter, J.P.N., Gregersen, N., Andresen, B.S. (2006). Short/branched-chain acyl-CoA dehydrogenase deficiency due to an IVS3+3A>G mutation that causes exon skipping. Human Genetics, 118 (6), pp. 680-690.
83. Schmidt, D.R., Hogh, B., Andersen, O., Hansen, S.H., Dalhoff, K., Petersen, E. (2006). Treatment of infants with congenital toxoplasmosis: Tolerability and plasma concentrations of sulfadiazine and pyrimethamine. European Journal of Pediatrics, 165 (1), pp. 19-25.
84. Skogstrand, K., Thorsen, P., Nørgaard-Pedersen, B., Schendel, D.E., Sørensen, L.C., Hougaard, D.M. (2005). Simultaneous measurement of 25 inflammatory markers and neurotrophins in neonatal dried blood spots by immunoassay with xMAP technology. Clinical Chemistry, 51 (10), pp. 1854-1866.
85. Johannsen, T.H., Mallet, D., Dige-Petersen, H., Müller, J., Main, K.M., Morel, Y., Forest, M.G. (2005). Delayed diagnosis of congenital adrenal hyperplasia with salt wasting due to type II 3β-hydroxysteroid dehydrogenase deficiency. Journal of Clinical Endocrinology and Metabolism, 90 (4), pp. 2076-2080.
86. Nielsen, H.V., Schmidt, D.R., Petersen, E. (2005). Diagnosis of congenital toxoplasmosis by two-dimensional immunoblot differentiation of mother and child immunoglobulin G profiles. Journal of Clinical Microbiology, 43 (2), pp. 711-715.
87. Meikle, P.J., Ranieri, E., Simonsen, H., Rozaklis, T., Ramsay, S.L., Whitfield, P.D., Fuller, M., Christensen, E., Skovby, F., Hopwood, J.J. (2004). Newborn screening for lysosomal storage disorders: Clinical evaluation of a two-tier strategy. Pediatrics, 114 (4), pp. 909-916.
88. Sørensen, T., Spenter, J., Jaliashvili, I., Christiansen, M., Nørgaard-Pedersen, B., Petersen, E. (2002). Automated time-resolved immunofluorometric assay for Toxoplasma gondii-specific IgM and IgA antibodies: Study of more than 130 000 filter-paper blood-spot samples from newborns. Clinical Chemistry, 48 (11), pp. 1981-1986.
89. Hjalgrim, L.L., Madsen, H.O., Melbye, M., Jørgensen, P., Christiansen, M., Andersen, M.T., Pallisgaard, N., Hokland, P., Clausen, N., Schmiegelow, K., Hjalgrim, H. (2002). Presence of clone-specific markers at birth in children with acute lymphoblastic leukaemia. British Journal of Cancer, 87 (9), pp. 994-999.
90. Jensen, U.G., Brandt, N.J., Christensen, E., Skovby, F., Nørgaard-Pedersen, B., Simonsen, H. (2001). Neonatal screening for galactosemia by quantitative analysis of hexose monophosphates using tandem mass spectrometry: A retrospective study. Clinical Chemistry, 47 (8), pp. 1364-1372.
91. Santer, R., Kinner, M., Steuerwald, U., Kjærgaard, S., Skovby, F., Simonsen, H., Shaiu, W.-L., Chen, Y.-T., Schneppenheim, R., Schaub, J. (2001). Molecular genetic basis and prevalence of glycogen storage disease type IIIA in the Faroe Islands. European Journal of Human Genetics, 9 (5), pp. 388-391.
92. Larsen, L.A., Armstrong, J.S.M., Grønskov, K., Hjalgrim, H., Macpherson, J.N., Brøndum-Nielsen, K., Hasholt, L., Nørgaard-Pedersen, B., Vuust, J. (2000). Haplotype and AGG-interspersion analysis of FMR1 (CGG)(n) alleles in the Danish population: Implications for multiple mutational pathways towards fragile X alleles. American Journal of Medical Genetics, 93 (2), pp. 99-106.
93. Gaustadnes, M., Rüdiger, N., Moøller, J., Rasmussen, K., Bjerregaard Larsen, T, Ingerslev, J. (1999). Thrombophilic predisposition in stroke and venous thromboembolism in Danish patients. Blood Coagulation and Fibrinolysis, 10 (5), pp. 251-259.
94. Lebech, M., Andersen, O., Christensen, N.C., Werte, J., Nielsen, W.E., Peitersen, B., Rechnitzer, C., Larsen, S.O., Nørgaard-Pedersen, B., Petersen, E. (1999). Feasibility of neonatal screening for toxoplasma infection in the absence of prenatal treatment. Lancet, 353 (9167), pp. 1834-1837.
95. Merryweather-Clarke, A.T., Simonsen, H., Shearman, J.D., Pointon, J.J., Nørgaard-Pedersen, B., Robson, K.J.H. (1999). A retrospective anonymous pilot study in screening newborns for HFE mutations in Scandinavian populations. Human Mutation, 13 (2), pp. 154-159.
96. Larsen, T.B., Lassen, J.F., Brandslund, I., Byriel, L., Petersen, G.B., Nørgaard-Pedersen, B. (1998). The Arg506Gln mutation (FV Leiden) among a cohort of 4188 unselected Danish newborns. Thrombosis Research, 89 (5), pp. 211-215.
97. Jiang, M., Aittomäki, K., Nilsson, C., Pakarinen, P., Iitiä, A., Torresani, T., Simonsen, H., Goh, V., Pettersson, K., De La Chapelle, A., Huhtaniemi, I. (1998). The frequency of an inactivating point mutation (566C → T) of the human follicle-stimulating hormone receptor gene in four populations using allele-specific hybridization and time-resolved fluorometry. Journal of Clinical Endocrinology and Metabolism, 83 (12), pp. 4338-4343.
98. Larsen, L.A., Grønskov, K., Nørgaard-Pedersen, B., Brøndum-Nielsen, K., Hasholt, L., Vuust, J. (1997). High-throughput analysis of Fragile X (CGG)(n) alleles in the normal and premutation range by PCR amplification and automated capillary electrophoresis. Human Genetics, 100 (5-6), pp. 564-568.
99. Eaton, R.B., Petersen, E., Seppänen, H., Tuuminen, T. (1996). Multicenter evaluation of a fluorometric enzyme immunocapture assay to detect toxoplasma-specific immunoglobulin M in dried blood filter paper specimens from newborns. Journal of Clinical Microbiology, 34 (12), pp. 3147-3150.
100. Lebech, M., Petersen, E. (1995). Detection by enzyme immunosorbent assay of toxoplasma gondii igg antibodies in dried blood spots on PKU-filter paper from newborns. Scandinavian Journal of Infectious Diseases, 27 (3), pp. 259-263.
101. Hansen, P.S., Nørgaard-Petersen, B., Meinertz, H., Jensen, H.K., Hansen, A.B.B., Klausen, I.C., Gerdes, L.U., Faergeman, O. (1994). Incidence of the apolipoprotein B-3500 mutation in Denmark. Clinica Chimica Acta, 230 (1), pp. 101-104.
102. Nørgaard-Pedersen, B., Høgdall, E.V., Arends, J., Vuust, J. (1994). Screening of newborn infants for cystic fibrosis. A combined analysis of immunoreactive trypsin and delta F508 mutation--a screening without false positive results [Screening af nyfødte for cystisk fibrose. En kombineret analyse af immunreaktivt trypsin og delta F508-mutationen--screening uden falsk positive.]. Ugeskrift for Laeger, 156 (25), pp. 3757-3760.
103. Lindhard, A., Græm, N., Skovby, F., Jeppesen, D. (1993). Postmortem findings and prenatal diagnosis of Zellweger syndrome. APMIS, 101 (1-6), pp. 226-228.
104. Gregersen, N., Blakemore, A.I.F., Winter, V., Andresen, B., Kølvraa, S., Bolund, L., Curtis, D., Engel, P.C. (1991). Specific diagnosis of medium-chain acyl-CoA dehydrogenase (MCAD) deficiency in dried blood spots by a polymerase chain reaction (PCR) assay detecting a point-mutation (G985) in the MCAD gene. Clinica Chimica Acta, 203 (1), pp. 23-34.
